# United Nations Convention on the Rights of Person with Disabilities (UNCRPD) Implementation: Perspectives of Persons with Disabilities in Namibia

**DOI:** 10.1155/2021/6693141

**Published:** 2021-05-26

**Authors:** Gwarega Chibaya, Pragashnie Govender, Deshini Naidoo

**Affiliations:** Discipline of Occupational Therapy, School of Health Sciences, University of KwaZulu-Natal (Westville Campus), Private Bag x54001, Durban 4000, South Africa

## Abstract

The Namibian government ratified the UNCRPD and its optional protocol in 2007 raising expectations that such a convention would fundamentally improve the lives of persons with disabilities. However, persons with disabilities continue to experience inequalities and violation of dignity. This study explores the impact of the UNCRPD as reflected on the lives of persons with disabilities in Namibia. An exploratory qualitative study with the use of photovoice and in-depth interviews was conducted in Omusati and Khomas regions, Namibia. Persons with disabilities (*n* = 31) were recruited via purposive sampling, of which *n* = 25 participants were engaged in three focus group discussions. Participants employed in the disability sector (*n* = 6) were engaged in in-depth interviews. Data were thematically analysed. The study findings revealed the inadequacy of disability rights information dissemination and continued barriers to inclusivity of persons with disabilities. Stigma, discrimination, limited financial opportunities, weak political support, and limited accessibility to physical infrastructure caused barriers to inclusivity. However, opportunities to advance the UNCRPD were also identified. There is a need for the disability sector to build on identified institutional facilitators to advance disability rights through mobilisation of local resources, communities, and government to redress the challenges identified in Namibia.

## 1. Introduction

Persons with disabilities constitute approximately 15% of the world population [1]. Africa has an estimated 60-80 million persons with disabilities [2]. In Namibia, persons with disabilities make up about 5% (98,413) of its population [3], while the World Health Organization (WHO) and World Bank estimates are at 15% (UNFPA 2018). According to the *World Report on Disability* [1], many persons with disabilities do not have equal access to health care, education, and employment opportunities; some are subjected to violence and prejudice. Denial of autonomy, violation of dignity, or inequalities experienced by persons with disabilities make disability a human rights issue that the United Nations Convention on the Rights of Persons with disabilities (UNCRPD) seeks to redress.

The Namibian government ratified the UNCRPD and its optional protocol in 2007. The UNCRPD creates an environment that promotes sustainable development goals 4 and 8 which advocate for inclusive learning environments and inclusive economic growth and accessible job markets for persons with disabilities, respectively [4]. In addition, it creates favourable conditions to enhance occupational justice and positive occupational outcomes. Occupational justice is seen to be consistent with the rights-based focus advocated by the persons with disabilities and disability rights activists, expressed by the UNCRPD as well as affirmed by the World Federation of Occupational Therapists' position on the centrality of occupation to health, well-being, and human rights [5]. The UNCRPD complements the occupational justice framework by addressing physical and situational barriers that create occupational injustices for persons with disabilities and promoting participation of persons with disabilities in occupations of their choice. In spite of embracing the UNCRPD, Namibia has limited detailed disability statistics and knowledge on challenges faced by persons with disabilities [3], which compromises the government's position to budget for disability-inclusive programmes. Negative attitudes and prejudice against persons with disabilities in Namibia affect their ability to participate in socioeconomic activities, including where they live and with whom one chooses to marry and start a family and to move about freely within the community [6, 7]. About 82.1% of children with a disability in rural and 17.9% in urban areas, aged five years and above, had never attended school [8].

According to Chichaya et al. [9], persons with disabilities experience occupational marginalization, occupational deprivation, occupational alienation, and occupational inconsideration. The occupational injustice experienced by persons with disabilities in Namibia is rooted in disability policy gaps [9] and possibly policy implementation challenges. For example, children with disabilities in Namibia attending school face many difficulties linked to the implementation challenges of the curriculum, attitudinal barriers, inaccessible infrastructure, and lack of access to appropriate teaching and learning technologies [8, 10]. According to the labour force survey, persons with disabilities form 9% (40,442) of the total inactive population of working age in Namibia [3]. This may be due to a lack of understanding of the need to invest in persons with disabilities by families, the business community, and country at large [10]. Possibly, all these could point to the reason why there are many barriers as well as widespread discriminatory attitudes that limit the participation of persons with disabilities in socioeconomic development activities in Namibia [1, 11, 12].

Policy implementation culture in Namibia depends on political control, procedures, and historical background. There are bottlenecks in the formalisation process of implementation, monitoring and evaluating policies in Namibia [13]. This delays the process of integration of international instruments into national legislation and development plans [14]. For example, the current, but dated, local National Disability Policy of 1997 needs substantial revision and overhaul [15].

The impact of the UNCRPD has not been documented in Namibia. However, the fact is that many persons with disabilities do not have access to different opportunities in Namibia [16]. This study is positioned to explore the influence of the UNCRPD on the lives of the persons with disabilities, their families, and their environment [17] in Namibia. Furthermore, it is aimed at gathering evidence on the understanding of the UNCRPD by persons with disabilities based on their lived experiences. More specifically, the aims of the study are (i) to explore on the awareness and knowledge of persons with disabilities on the purpose and contents of local policies/guidelines/circulars and UNCRPD, (ii) to identify the perceptions of persons with disabilities on actions implemented by the government as part of enforcing the UNCRPD, and (iii) to determine the facilitators and barriers to enjoyment of rights among persons with disabilities in Namibia.

## 2. Material and Methods

### 2.1. Study Design

The study followed an explorative qualitative approach using photovoice as a visual method, focus group discussions, and in-depth interviews to collect data. With the use of photovoice, persons with disabilities used cameras to capture images of situations that reflected their day-to-day activities. Focus group discussions were used to collect data from persons with disabilities through group interaction. The in-depth interviews were used to explore the opinion of the key informants who were persons with disabilities who held positions of authority in the disability sector. The research problem was framed and analysed through a transformative lens in which the inquiry is intertwined within an action agenda for reform or changing lives of the participants, through confronting social oppression at all levels [18].

### 2.2. Study Setting

The study was conducted in Namibia. Participants were recruited from the Omusati region, which has the largest number (15,230) of persons with disabilities and the Khomas region with a population of 10,713, which is centrally located within the capital city of Namibia (Windhoek) and which houses several organisations coordinating disability activities in the country [3]. Additionally, the Khomas region recorded 45% inflow of persons with disabilities from all other regions [2].

### 2.3. Study Population and Sampling

Nonprobability, purposive maximum variation sampling was used to recruit participants who were persons with disabilities from the two regions. Purposive sampling is a strategy where the researcher chooses participants based on his judgement on who can provide information to achieve the objectives of the research [19]. Participants were selected based on their knowledge and exposure to the UNCRPD from the identified population. Snowball sampling was incorporated to recruit more persons with disabilities, where the sampled participants provided referrals of other potential participants, who were contacted and recruited for participation. Each identified participant presented a health card to confirm their type of impairment.

Thirty-one participants participated in this study stratified by several characteristics (Tables 1–4). These participants used their personal experiences and understanding to describe their perceptions on the implementation of the UNCRPD and the local disability policy documents in advancing disability rights in Namibia. Twenty-five participants were engaged in photovoice discussions (Tables 1–3), and six participants were interviewed (Table 4). The majority of the participants (*n* = 25) were not employed, while only six participants had occupations within the disability sector and became the key informants.

### 2.4. Data Collection

Data were collected using photovoice, focus group discussion, and in-depth interviews. Research assistants were used to assist with the translation and sign language during focus group discussions. Data were collected until no new information emerged from the participants. The research assistants were qualified social workers and were trained on the subject of discussion. Data were recorded on a digital voice recorder and manually transcribed while the pictures were stored in a password-protected computer with consent from each participant.

#### 2.4.1. Photovoice

Photovoice is a visual research method used in qualitative research where participants take photographs during their day-to-day lives, which help them to capture their emotions, ideas, and thoughts about a phenomenon, and then share these photographs in a focus group discussion, reflecting upon these photos of their lived experiences [20]. Participants were requested to take photographs that reflected their community experience (strengths or concerns). Each participant was given a camera and instructions to follow. The participants took pictures which they used within the focus group discussions. Twenty-five participants took pictures that explained the strength or challenges they were faced within their everyday environment. They were engaged in three separate photovoice discussions. The images captured by the participants became the central artefacts in conveying the meaning and perceptions and provoking critical engagement during focused group discussions [21]. This method was valuable towards gaining insight into some otherwise hidden aspects of the individual's daily life experience and needs. The photographs elicited and promoted critical dialogue during the focus group discussion on the implementation of the UNCRPD from the participants' perspective.

#### 2.4.2. Focus Group Discussion

This data collection method involved persons with disabilities, who were not employed during the time of the research, sharing their perspective on the implementation of the UNCRPD as reflected in their daily lives. Three focus group discussions were conducted, with each constituting eight to nine persons with disabilities (Tables 1–4). Each group constituted participants with diverse impairments. Each group started with a discussion of the photographs that elicited a critical discussion led by the principal author. Participants were probed to stimulate further discussion with the participants. The discussions were audiorecorded and photographs shared with the principal author.

#### 2.4.3. In-Depth Interviews

The persons with disabilities who were employed within the disability sector were engaged in face-to-face in-depth interviews rather than focused group discussions. Only six persons with disabilities were employed within the disability sector and were recruited as participants in this study. The interview choice allowed for a relaxed environment that enabled discussion of their perspectives without fear of those (other persons with disabilities) whom they are intended to serve. The participants in this category chose their place of work for interviews. The interviews were audiorecorded.

### 2.5. Trustworthiness and Ethical Considerations

Transgressive validity was applied to this study, which stimulates thinking about how knowledge is created and evaluated and whether the research accomplishes its goals it intended to achieve. The principal author engaged the available literature on the implementation of the UNCRPD in Africa and Namibia prior to and during the study to gain insight and richness into exploring the research objective. Given that the principal author works within the disability sector as a policy implementer, reflexivity was exercised to suspend judgements via journaling and peer debriefing. In addition, triangulation was ensured through the use of multiple methods and sources of data [22]. The participants from different regions had different types of impairments, and some participants who held positions of authority in various organisations were included. This enabled maximum variation from a diverse group of participants. The questioning approach of the first author and research assistant remained consistent to each member in the group and each group of participants to ensure dependability. However, transferability will depend on contextual similarities. Following the development of the themes, research participants were engaged to determine if these themes were representing their views (member checking/respondent validation). The University of KwaZulu-Natal Biomedical Research Ethics Committee (reference number BE022/19) and the Namibia Ministry of Health and Social Services Research Management Committee (reference 17/3/3GC) granted ethical approval for this study. Ethical principles were upheld, where written informed consent was obtained from the participants and confidentiality and anonymity were preserved.

### 2.6. Data Analysis

Data analysis methods are procedures for manipulating data so that the research question can be answered, usually by identifying significant patterns [23].

#### 2.6.1. Photovoice

The principal author recognised narratives and photographs as meaningful data depicting the participants' lived experience. The analysis of the photographs consisted of four stages: a photograph analysis based on the researcher's interpretations, a photograph analysis based on the participants' interpretations, a crosscomparison, and theorisation [24]. The photographs were reviewed from the authors' perspective independent of the participants' interpretations to develop a preliminary understanding. The photographs were categorised and recategorised until there was no new information emerging. Preliminary themes were built from the authors' interpretation. This was followed by an analysis of the photographs based on the participants' interpretations to determine alternate explanations. The same process of categorisation and recategorisation of both the photographs and narratives into themes continued until saturation. A cross comparison of the findings from these two processes was done. Similar themes were integrated. The final stage focused on generating visual and narrative representations based on themes developed during the crosscomparison.

#### 2.6.2. In-Depth Interview and Focused Group Discussion

**Figure 1:**
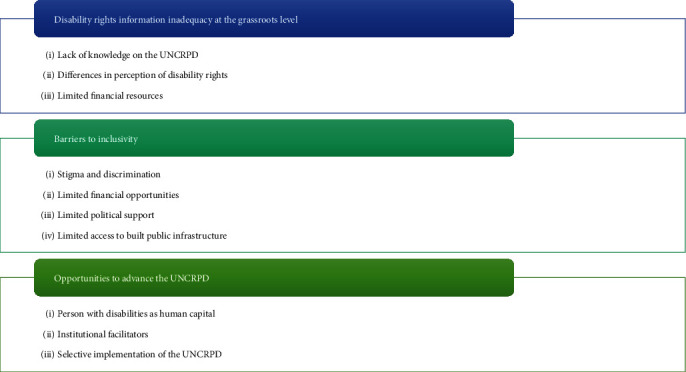
Themes and categories.

The in-depth interviews and focused group discussions were transcribed into written form to enable thematic analysis. After reading and rereading the individual transcripts for familiarity with the data sets, the principal author constructed codes, which were unified and organised into categories. The categories from in-depth interviews were combined, and some merged with the categories obtained from the photovoice. These categories were unified and organised into themes (Figure 1), which merged from the perceptions and experiences shared by persons with disabilities in photovoice discussions and in-depth interviews. The codes and the themes were discussed, reviewed, and refined by the coauthors. Verbatim quotations and photos taken by persons with disabilities were used to present evidence of the participants' perceptions and experiences. Patterns and themes were identified.

## 3. Results

### 3.1. Theme 1: Disability Rights Information Inadequacy at the Grassroots Level

The theme, disability rights information inadequacy at the grassroots level, is discussed under three categories: lack of knowledge on the UNCRPD, differences in perception of disability rights, and limited resources.

#### 3.1.1. Lack of Knowledge on the UNCRPD

This category yielded sentiments that suggested a lack of awareness of the purpose and contents of the UNCRPD and local disability policy documents. A total of 21 participants did not know the UNCRPD, its contents, and any other disability policy documents in Namibia. These participants did not hold any position of authority in the disability sector. Six participants (who held a position of power in the disability sector) were aware of the UNCRPD, its contents, and local disability policy document. Still, they had severe misconceptions about its use to defend persons with disabilities from violating their rights. For example, all the participants felt that persons with disabilities would not win a litigation case against persons without disabilities who violate their rights. Persons with disabilities do not understand that the UNCRPD is a tool that empowers them to advance their rights or hold anyone who violates their rights to account.

*People do not know their rights and the UNCRPD. Every time you will bring these documents (UNCRPD and other policies) to people, they will say this is new to them. (KI3).*

#### 3.1.2. Differences in Perceptions of Disability Rights

This category explains the identified differences in perceptions among persons with disabilities who participated in the study. There is no unified voice on what is needed by persons with disabilities from those in positions of authority in the disability sector and those without the position of power. The source of such differences comes from a broader gap in knowledge of the UNCRPD. The perceptions of participants who held positions of authority in the disability sector on the disability rights discourse were influenced by their understanding of the UNCRPD due to exposure to disability rights documents within their offices. These participants work in the offices dealing with disability issues daily. Hence, their focus is on rights advancement, as indicated by some key informants.

*Yes, that (UNCRPD) is our bible. That is what we preach about in Namibia especially me. If you ask me articles in the UNCRPD, I know what is in it … What we are advocating for is for us as persons with disabilities to participate in the decision making process because we realise that is where the main problem is, because we are not part of the decision making body. (KI2).*

In contrast, 25 of the participants (who did not hold any position of authority in the disability sector) perceived disability rights to be rooted in a charitable model of disability through which they articulated their understanding of their rights. These participants have not been exposed to the UNCRPD. They view the lack of donations and help as the source of their suffering, as indicated in their narrative below.

*In Oshana region, persons with disabilities received good help like wheelchairs, walking cane and other materials. In our region (Omusati), we buy ourselves. We are asking our government to give us the wheelchairs freely. (OM6).*

#### 3.1.3. Limited Financial Resources

All participants identified limited financial resource as the leading cause of the challenges the persons with disabilities experience in Namibia. The persons with disabilities who held positions of authority could not conduct awareness programmes due to lack of funds. In addition, availed donor funds were meant for specific donor activities, which were not prioritised as necessary within the Namibian context. The persons with disabilities who did not hold any positions complained of a lack of appropriate wheelchairs and assistive devices in public hospitals due to limited funds allocated for disability activities. One of the participants narrated the mismatch of resources.

*I think we are not being realistic on the issues of disability, you know it is very expensive and the money that is being put into it is like not even a quarter of what is needed. We are asking this and was supposed to be proven in terms of value, how much money do you actually give to facilitate disability activities, for example to empower the citizens with disabilities, how much money is actually needed, annually, how much is it? (KI4).*

### 3.2. Theme 2: Barriers to Inclusivity

This theme focuses on identified barriers to the inclusion of persons with disabilities in different activities that promote rights and development. Stigma and discrimination, limited financial opportunities, limited political support, and limited accessibility of physical infrastructure were categorised as causes of barriers to inclusivity of persons with disabilities.

#### 3.2.1. Stigma and Discrimination

Stigma and discrimination have subjected persons with disabilities to isolation, marginalisation, and loneliness, resulting in abuse, violence, neglect, labelling, ignorance, and fear. All participants experienced stigma and discrimination from family or community members. For example, parents may deny children with disabilities the right to outdoor traditional play activities since they fear that they will suffer in their absence. Most participants agreed that such fears are justifiable given that the environment is not accessible, and there are no assistive devices needed to improve independence in activities of daily living. The majority of the participants cannot easily access leisure and recreational activities. Participants reported that family members do not have time and patience for persons with disabilities. Stigma and discrimination can occur without the knowledge of the perpetrator, as narrated by the participants.

*You might find also in our houses that parents or family members discriminating a person with disability while they believe they are doing a good thing for the person and the person himself really think they are doing well for him. For example, they say you do not need to go to school because you cannot walk, it is difficult for you, because they believe that our child will be suffering at school which is true in most cases and the person with disability believe that I am going to suffer. (KI2).*

Discriminated participants experienced a downward spiral ending in a web of further discrimination due to their desire to survive. For example, the intimacy relationships among persons with hearing and speech impairments are limited due to the belief that they need to have relationships/socialise with people who have hearing and speech impairments. This will enable them to retain their ability to use sign language. This unfortunate dynamic process has been narrated as follows.

*Sometimes they do not help to interpret what they are talking, and I become very angry. Sometimes when they are talking, laughing and looking at me I think they are gossiping me. It is hard because I cannot hear anything and I cannot be a part of the conversation. We always look for people that are like us. With deaf people, you are able to communicate and if we do not communicate with people that are speaking sign language, your signing abilities go away. (KHR5).*

#### 3.2.2. Limited Financial Opportunities

Financial opportunities are not readily available to persons with disabilities. Persons with disabilities complained that banks are reluctant to finance their small businesses or development based on using the disability pension money as security for repayment. There are limited employment opportunities. A total of 25 participants did not have minimum academic requirements for employment. Even if the qualification requirements for work were reduced, helpless employers' attitudes and the environment without reasonable accommodation would preclude persons with disabilities from employment from earning a decent salary.

*If you call me to an interview and yet in your advertisement, you said persons with disabilities are encouraged to apply, but then your building is not conducive for me to enter. I will end up at the doorstep. I am hearing impaired and in your interview panel, there is no one who can do sign language. You are excluding persons with disabilities by doing that. (KI1).*

The government issued a directive to all employers to include a clause that persons with disabilities are encouraged to apply for any advertised post as alluded to by six participants. There have been noted efforts by employers to add such a clause on many advertisements. However, it was perceived as a practice aimed more at appeasing the government than actually to include persons with disabilities in work; hence, persons with disabilities remain largely excluded from the labour force.

#### 3.2.3. Weak Political Support

Six of the participants felt that the politicians ignore disability issues. All disability matters have been pushed to a single ministry. This makes it difficult for disability issues to be integrated into other ministries. Six participants had a strong feeling that the politicians do not understand issues surrounding disability, which makes it difficult to defend the needs of persons with disabilities. This contributed to the delays in writing the progress report on the implementation of UNCRPD. What all participants viewed as lack of a political will can be seen to some extent as a lack of knowledge on the UNCRPD by the politicians. However, it remains clear that there is this lack of political will as stated by one key informant participant.

*I find that fellow politicians do not give much on issues of disability. You find it rare for disability issues to be mentioned in parliament in terms of Human Rights or development of persons with disabilities. You will hear often here and there someone will talk about disability in a charitable way, not really in a robust developmental way that we want. (KI1).*

#### 3.2.4. Limited Access to Built Public Infrastructure

Most public buildings in towns have included accessibility within Namibia, with marked limited accessibility to commercial buildings in high-density areas. However, concerns were raised by the key informants concerning standards of accommodation of buildings and understanding of accessibility of infrastructure to persons with different disabilities. There is no clear understanding of accessibility as illustrated in the following voice.

*Some think making a building accessible means making a ramp only for a wheelchair to get in the building. Making the building accessible requires a lot of things, you need to take the need of visually impaired and you need to take care of the needs of the deaf persons and you need to take care of the wheelchair users including the bathroom and all those things should be accessible. Although we have the standard, they say that the requirement of the local government act that all the buildings should be accessible but the architect comes with their own understanding of accessible and will design the way he thinks. The builder when he comes he will build according to the building plan. (KI2).*

**Figure 2:**
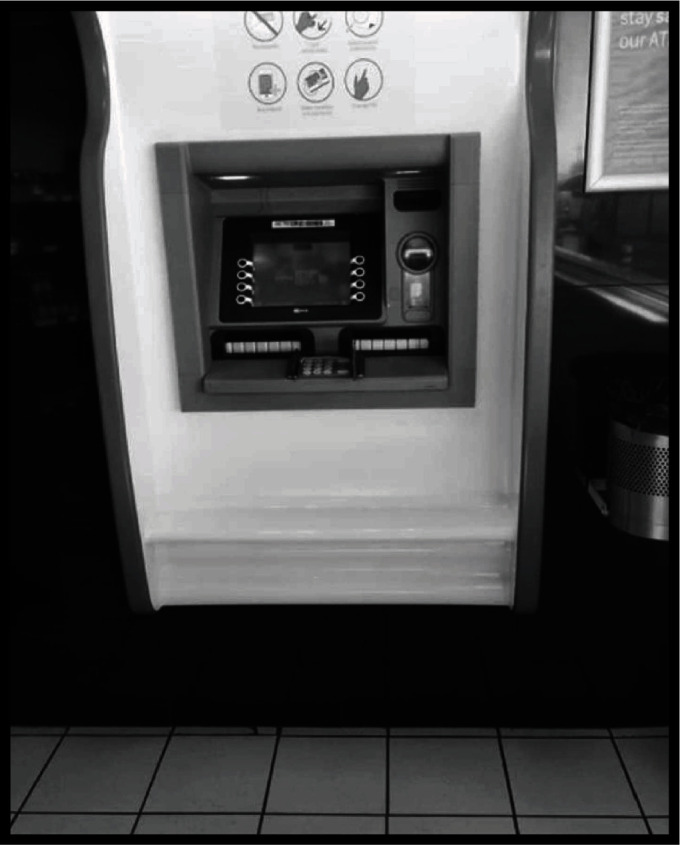
Automated teller machine that is not user friendly to persons with visual impairment (figure recoloured to prevent identification of the service provider).

Some infrastructures do not cater to other types of disabilities apart from wheelchair users. One of the participants (KH6) in the Khomas region captured the automated teller machine to illustrate her voice on how persons with visual impairment struggle due to lack of audioservices (Figure 2).

The challenges with infrastructure have been noticed along main tarred roads as well. About six of the participants felt that persons with visual impairments were not considered during infrastructure development.

*Our roads are not yet up to standard. Let us say our robots (traffic lights), it is still more for people with a normal vision. Us the visually impaired cannot stop and tell what does the noise from the robot (traffic light) means apart from knowing that there is a robot. The sound does not change whether the robot (traffic lights) is green or red. It cannot tell whether now it is my turn to cross the road and so. People did not understand the concept of the sound of our robots (traffic lights). (KH5).*

**Figure 3:**
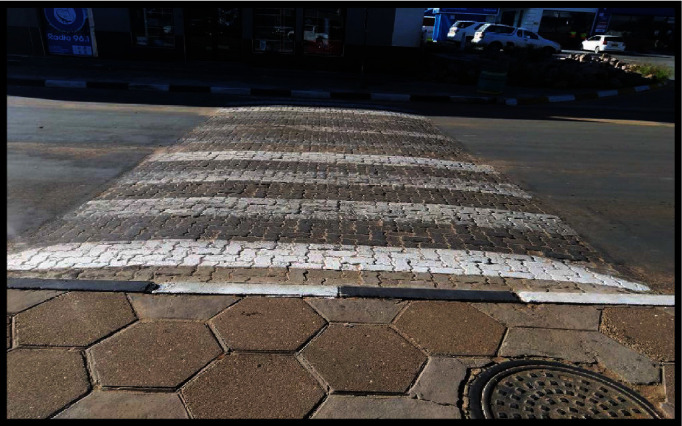
Pedestrian crossing place that lacks inclusivity for wheelchair users.

The challenges with the construction of infrastructure were further supported by the illustrated voice (KH6) in Figure 3.

Persons with disabilities on a wheelchair struggle to navigate across the roads at pedestrian crossing lines without assistance due to lack of continuation of the hump (Figure 3) at the crossing place thereby creating a barrier.

Besides, old infrastructure buildings were reported by all the participants as having accessibility challenges. Most of the buildings with stairs and lifts are being used as government offices and schools in Namibia. The elevators constantly malfunction, making it difficult for persons using wheelchairs to access services on other floors.

*Go into the building, most of the building were unfortunately built before people began to think about accessibility, they have stairs, if they have lifts, the lifts are constantly broken. (K13).*

**Figure 4:**
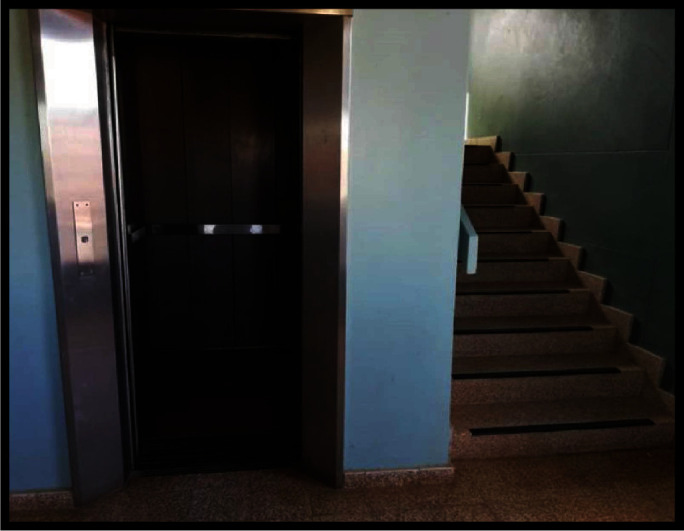
Five-story building accessible through a lift and stairs only.

The same view was illustrated with a photo as to how such buildings pose a challenge to persons with lower limb impairments when elevators are not functional.  Figure 4 demonstrates the only available options for access into the building; therefore, wheelchair users have only one option to access the building.

**Figure 5:**
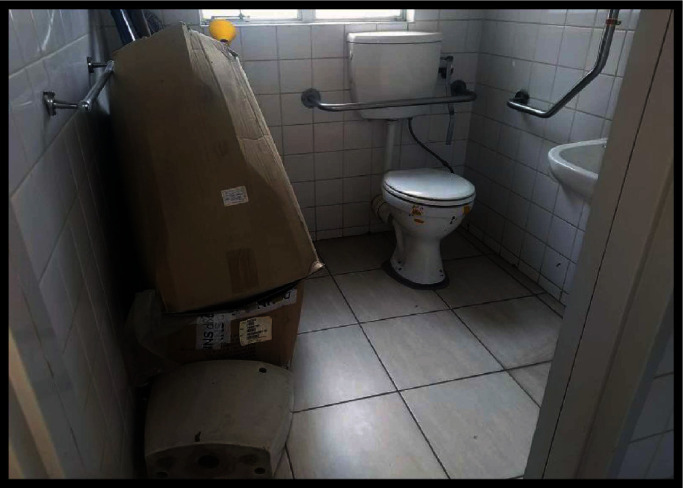
A toilet for wheelchair users used as a storage room.

**Figure 6:**
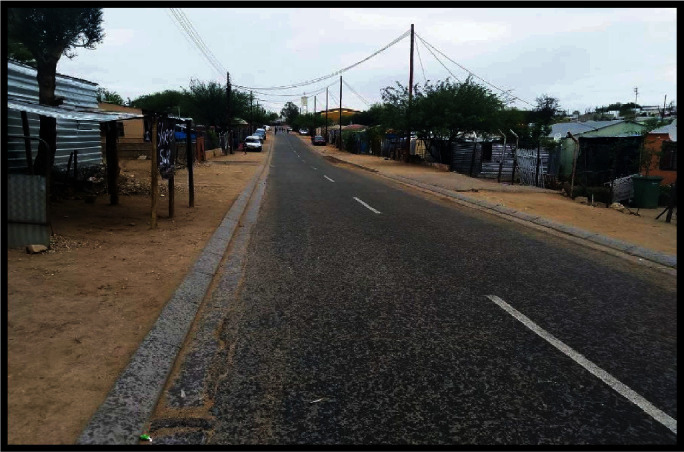
A picture illustrating unauthorised extended building/cars covering available space/sidewalks for wheelchair mobility (or no pavements for wheelchairs).

Five participants appreciated the limited progress in improving accessibility, especially for wheelchair users in large shops. It was however noted that human activity, attitude, and behaviour sometimes hinder indoor or outdoor accessibility as illustrated through the photo illustrations (Figures 5 and 6).

In Figure 5, a toilet captured in the Khomas region, which is wheelchair-friendly, is being used as a storage room. The participants concurred that people are not aware of the barriers they create for persons with disabilities each time they disregard the value of having these dedicated facilities for persons with disabilities.

Outdoor barriers caused by unapproved constructed structures and unpaved roadsides limit space for wheelchair mobility, as shown in Figure 6.

Contrary to challenges experienced in the town, seventeen of the participants from villages and periurban areas complained of problems caused by the environmental terrain, which poses a greater danger to them and their assistive devices and wheelchairs. One of the female participants highlighted the following:

*Persons with disabilities are better there in town. It is better for them. (OM2).*

**Figure 7:**
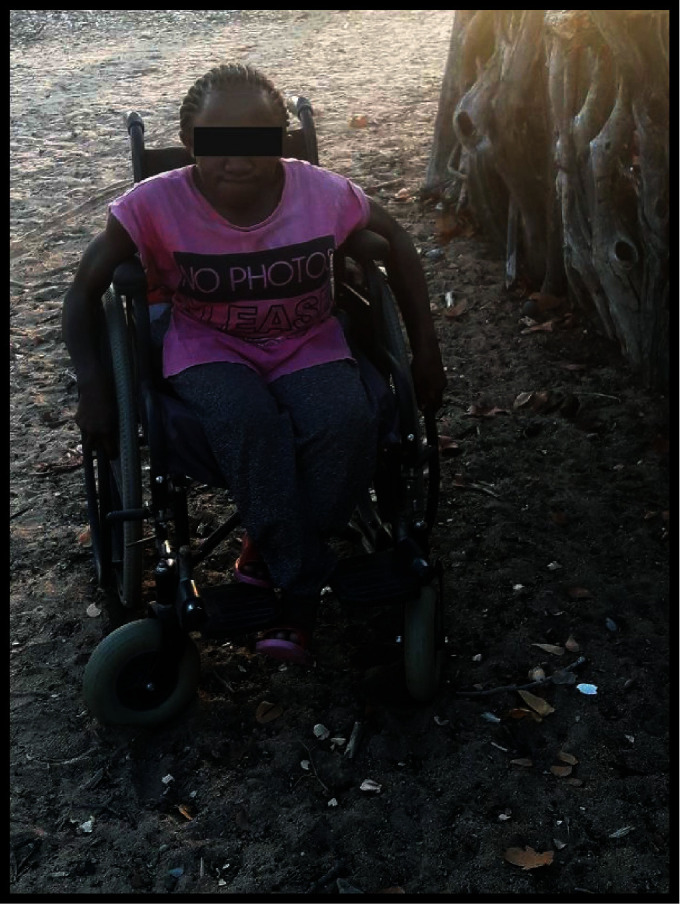
A wheelchair not suitable for sandy terrain.

**Figure 8:**
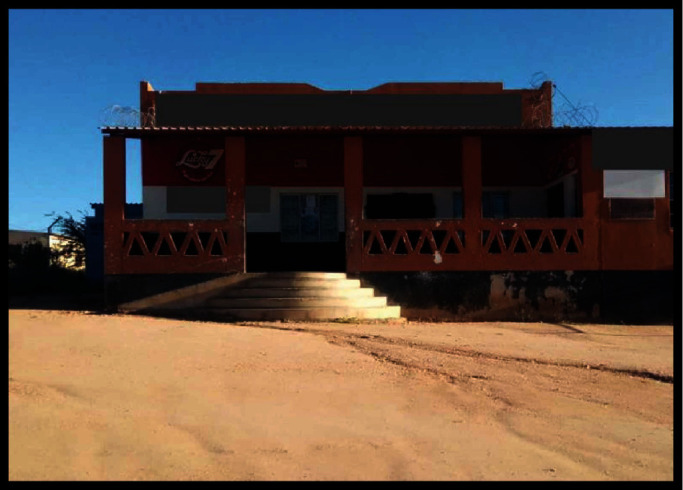
Community butchery with no accessibility to wheelchair users and people with mobile walkers.

This narrative has been demonstrated through photo illustration (Figures 7 and 8) showing how both the terrain and infrastructure are difficult to access in villages.

Challenges posed by the terrain in the villages (Figure 7) and inaccessible buildings (Figure 8) as illustrated voices support the idea that more challenges exist in villages.

### 3.3. Theme 3: Opportunities to Advance UNCRPD

This theme covers three categories: persons with disabilities as human capital, selective implementation of the UNCRPD, and institutional enablers.

#### 3.3.1. Person with Disabilities as Human Capital

Persons with disabilities demonstrated some talents and skills that they are using to earn a living despite challenges experienced in their environment. Some are entrepreneurs, in businesses selling food, while others are involved in fashion and designing, among other things. A few of the individuals secured positions in the disability sector and may be resourceful to their counterparts in championing disability rights discourse. Their reflection was an indication of what persons with disabilities can do if they are empowered.

*Like me, I got training from the National Disability Council, and with National Youth Council on writing projects, and how to manage a project for a certain business. Especially now, I am very happy for the course that is being given by NDCN, the business course, this is the course that helped me. For example, I run my own business because of the knowledge that I got from that course. It does not help if we are not explaining these things or taking these things into greater levels. I run a shop. (KI4).*

**Figure 9:**
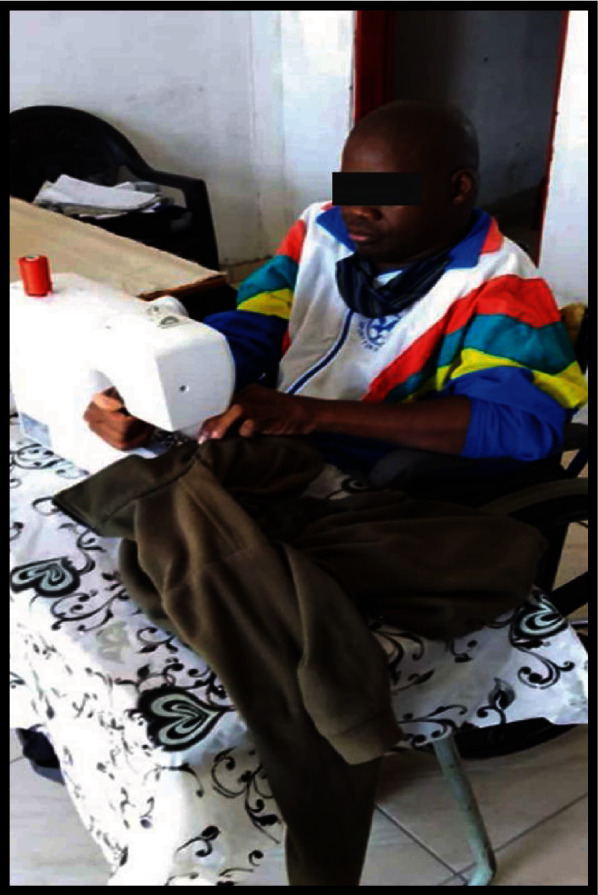
A tailor on a wheelchair sewing clothes.

This category found resonance with an illustrated voice (Figure 9).

Figure 9 describes one of the talents and skills possessed by the participant. Despite using a wheelchair and challenges experienced to access his workplace due to sand terrain, the participant goes to design and sew clothes daily to earn a living.

#### 3.3.2. Institutional Enablers

There were notable institutional enablers that can foster and support the acceleration of the implementation of the UNCRPD in Namibia. Namibia's articles 143 and 144 of its constitution make the signed UNCRPD part of the domestic laws since its ratification had no reservations [23]. Namibia has the National Disability Council, which monitors disability rights implementation and violation. These strengthen the foundations for advancing disability rights. Persons with disabilities who held positions of authority identified these institutional facilitators.

*The President has taken to establish an office dealing entirely with disability issues under the auspice of the Vice-President. Currently we amending the National Disability Council Act and National Disability Policy to bring them in line with the UNCRPD*. *We are going every year to the UNCRPD international conferences.* (KI1).

#### 3.3.3. Selective Implementation of the UNCRPD

The UNCRPD has been sparingly put into action in Namibia, as indicated in some identified activities. The participants who held positions of authority recognised the adoption of inclusive education even though the country remains with a strong tradition of segregated schooling. The government has amended policies to safeguard the employment of persons with disabilities and provides monthly grants to persons with disabilities.

The recruitment of specialised foreign expatriates in the rehabilitation departments and the provision of wheelchairs and assistive devices were alluded to by five participants. However, misunderstandings on how to enforce implementation, lack of mechanisms for monitoring, and absence of a transparent aggregated database on the number of persons with disabilities in the country were reported.

*I received cases from schools of children referred by the teachers to go to special schools. If you ask the teacher where that special school is they have no idea. Therefore, people do not understand what a special school is, what inclusive education is and what the move of the government toward inclusive education is. Teachers who are in the class do not understand. You might find that in some schools even the principal will tell you I have heard about inclusive education but I do not agree with it (KI2).*

Therefore, lack of knowledge, unity of purpose, and misunderstanding on implementation of UNCRPD as noted by the participants speak to the confusion noted on the implementation strategies within the country.

## 4. Discussion

### 4.1. Key Findings

Inadequacy of disability rights information and existing barriers to the inclusion of persons with disabilities were identified as the leading cause of challenges experienced by persons with disabilities in Namibia. On the other hand, institutional enablers such as the President establishing an office dealing with disability issues under the vice-president's auspice were seen as potential avenues to advance disability rights [3].

### 4.2. Discussion of Key Findings

Inadequacy of disability rights information and barriers to inclusivity chronicles evidence that the communities in Namibia still need to adjust their culture and behaviour to accommodate persons with disabilities [25]. Persons with disabilities are not empowered with disability information required to challenge the daily occupational injustices experienced [26]. They cannot insist on their right to participate in the process of national development [17].

The persons with disabilities do not know their rights enshrined in the UNCRPD and the potential resources available for use, confirming previous findings [27, 28]. They lack knowledge of the strategies to follow when their rights are violated. The difficulties in accessing disability rights information by persons with disabilities have been documented in previous studies [27, 28]. Persons with disabilities in rural areas and periurban areas are more affected given that the resources and the OPDs are more concentrated in urban areas. Even though the resources are in urban areas, the availability of the UNCRPD and policies alone cannot improve disability rights without massive transfer of such information to persons with disabilities and the community. The gaps in disability rights knowledge could contribute to the vulnerability of persons with disabilities at a grassroots level in Namibia [27]. Therefore, the inadequacy of disability rights information could be linked to inequalities experienced by persons with disabilities, rather than inherent impairments reported in previous studies [12, 29, 30].

These gaps in disability rights knowledge prevented the participation of persons with disabilities in the domestication process of the UNCRPD [17], resulting in a lack of pressure exerted on the government and the offices of persons with disability (OPDs) to implement reforms in line with the UNCRPD as documented in a previous study [31]. For example, the delays by the government to reform the national disability policy and to write the initial country report on the implementation of the UNCRPD have been long overdue by 13 years.

There is a potential conflict of interest in terms of persons with disabilities working in OPDs to advance disability rights and holding the government to account while earning salaries from the same government. This concurs with findings from Mwendwa et al. [32]. The lack of progress toward transforming the situation of persons with disabilities, their families, and their environment has created indifference among persons with disabilities with and without a portfolio. These findings were aligned to a study that cited tensions in the disability rights movement due to contradictions in the approach to address disability rights challenges [33]. This jeopardises unity and the effectiveness of the demands of persons with disabilities.

Disability inclusion is creating an enabling environment supported by adequate policies and practices in which persons with disabilities participate freely, fulfilling family and community roles and responsibilities similar to their able-bodied peers [34]. Stigma and discrimination, inconsistent accessibility of infrastructure, limited financial opportunities, and limited political support contributed to limited disability inclusion in Namibia and mirrored previous studies [8, 35–41]. Moreover, there is a lack of a well-informed investment in persons with disabilities needed to enable sustainable development [42].

Minimal participation of persons with disabilities contributed to limited advancement opportunities for disability rights noted in Namibia. The government has been left alone deciding what to implement with insufficient financial resources and many other competing priorities indicated in previous studies [8, 29, 43, 44]. For example, the government provides a monthly disability pension grant that the persons with disabilities have complained to be too little to protect livelihoods or to be used as collateral security to access mainstream microfinance as documented in some studies [45, 46]. Evidence of entrepreneurship opportunities noted was limited to educated and employed persons with disabilities, hence the call for support in the inclusivity of persons with disabilities in education [8, 47]. Each additional year of schooling for persons with disabilities will improve opportunities to compete in entrepreneurship, and other markets were noted with decent wages, as indicated in studies done in China [48].

The progress of implementing the UNCRPD is slow, evidenced by the delayed country report on its domestication, unavailability of statistical data on the prevalence of persons with a disability, lack of data monitoring tools, and differences in understanding of what needs to be done. This appears to correspond well to the results presented in other studies [2, 29, 47, 49]. Besides, selective implementation of the UNCRPD has resulted in a lack of quantifiable impact on the effectiveness of the interventions employed [39].

### 4.3. Strengths and Limitations

This study focused on implementing the UNCRPD in Namibia based on living experiences and perceptions of persons with disabilities. The authors managed to gain insight into the gaps that contributed to implementation challenges of the UNCRPD, which hinders persons with disabilities from enjoying disability rights despite the government of Namibia's overall commitment to promoting disability rights. The research did not address individual articles in the UNCRPD. However, most challenges faced by persons with disabilities were related to lack of enforcement of specific articles, for example, articles on awareness-raising, accessibility, freedom from exploitation, violence and abuse, personal mobility, access to information, education, work and employment, adequate standard of living and social protection, statistics and data collection, and equal recognition before the law within the UNCRPD. Future researchers may look at the implementation of particular articles of the UNCRPD and find ways of addressing identified gaps through engaging the disability sector.

### 4.4. Implications and Recommendations

This study reinforces the need to strengthen and accelerate the promotion of the disability rights activities and awareness-raising of the UNCRPD by providing accurate information, which creates a positive impact on the lives of persons with disabilities [17, 50]. Building on the identified institutional facilitators, the OPDs should work with persons with disabilities to build links with leaders, local employers, the business community, schools, and microfinance institutions, among others [43]. This will address the inadequacy of disability information at the grassroots level. Tradeoffs can assist in resolving the differences among persons with disabilities [31]. Reforms on social and institutional norms and practices through robust disability rights debate promote disability inclusivity [50, 51]. Engagement and empowerment of persons with disabilities put the country on a path to achieve its vision 2030 and sustainable development goal, further contributing to the master plan of transforming Africa, Agenda 2063. Economic inclusivity of persons with disabilities will boost productivity raising the gross domestic product [42]. Inclusivity is cost-effective as compared to the selective implementation of the UNCRPD [52].

These revelations call for a robust engagement, debate, and discussion that stimulate the inclusion of persons with disabilities breaking barriers that perpetuate marginalisation. The principal author will engage the OPDs on the findings of this study. In turn, the OPDs will engage the persons with disabilities, policymakers, and other implementers on the findings and deliberate on how to address the identified challenges. The principal author, working together with some of the participants and different stakeholders within the disability sector, will work to develop a strategic intervention aimed at promoting the implementation of the UNCRPD.

## 5. Conclusions

This article has confirmed the inadequacy of disability rights information dissemination at the grassroots level in Namibia. In addition, barriers to inclusivity in accessing employment, mainstream financial support, and infrastructure have been confirmed as the source of challenges in mainstreaming persons with disabilities in community development initiatives at the grassroots level. On the other hand, the study has shown some institutional facilitators laid down by the government in favour of advancing disability rights. This includes the disability affairs focal office within the office of the vice-president, the availability of the National Disability Council of Namibia, a watchdog in monitoring and enforcement of disability rights, and the existing international partnership funding disability initiatives. Furthermore, the country has started mobilising resources to enable the amendment of the National Disability Policy of 1997. The milestone achieved in implementing some articles of the UNCRPD in education, employment, and health, including ensuring social protection of the persons with disability, is a significant commitment shown by the government.

## Figures and Tables

**Table 1:** Summarised description of participants with disabilities from the Omusati region that engaged in a focus group discussion (*n* = 8).

Participant ID	Gender	Age	Impairment type	Urban/rural	Occupation at the time of the study
OM1	F	36	Paraplegia	Rural	Unemployed
OM2	F	50	Paraplegia	Rural	Unemployed
OM3	F	30	Hearing impairment	Rural	Unemployed
OM4	F	35	Visual impairment	Rural	Unemployed
OM5	F	55	Congenital malformations	Urban	Unemployed
OM6	M	44	Paraplegia	Urban	Employed
OM7	M	44	Paraplegia	Urban	Unemployed
OM8	F	55	Diplegia	Rural	Unemployed

**Table 2:** Summarised description of participants with disabilities from the peri-urban Khomas region that engaged in a focus group discussion (*n* = 9).

Participant ID	Gender	Age	Impairment type	Urban/rural	Occupation at the time of the study
KHR1	M	24	Hearing impairment	Urban	Part-time
KHR2	M	34	Paraplegic	Urban	Employed
KHR3	M	26	Congenital malformations	Rural	Unemployed
KHR4	F	29	Lower limb amputation	Urban	Employed
KHR5	F	35	Hearing impairment	Urban	Unemployed
KHR6	F	29	Hearing impairment	Urban	Unemployed
KHR7	F	39	Visual impairment	Urban	Unemployed
KHR8	F	42	Visual impairment	Urban	Unemployed
KHR9	F	33	Unilateral lower limp amputation	Urban	Unemployed

**Table 3:** Summarised description of participants with disabilities from the urban area of Khomas region that engaged in a focus group discussion (*n* = 8).

Participant ID	Gender	Age	Impairment type	Urban/rural	Occupation at the time of the interview
KH1	M	49	Mental illness	Urban	Unemployed
KH2	M	30	Paraplegia	Urban	Unemployed
KH3	F	20	Learning disability	Urban	Unemployed
KH4	F	34	Bilateral amputation	Urban	Unemployed
KH5	F	39	Visual impairment	Urban	Unemployed
KH6	F	45	Paraplegia	Urban	Unemployed
KH7	M	33	Visual impairment	Urban	Unemployed
KH8	M	33	Hearing impairment	Urban	Unemployed

**Table 4:** Summarised description of key informants with disabilities interviewed (*n* = 6).

Participant ID	Gender	Urban/rural	Region	Occupation at the time of the interview
KI1	F	Urban	Khomas	Representative of disability affairs
KI2	M	Urban	Rundu	Representative of national federation of persons with disabilities
KI3	M	Urban	Khomas	Representative of the National Disability Council
KI4	M	Urban	Khomas	Representative of youth with disabilities
KI5	M	Urban	Omusati	Representative of women with disabilities
KI6	F	Urban	Khomas	Representative of organisations of persons with disabilities

## Data Availability

All data derived from this part of the study is presented in this manuscript.
